# Integrating diverse biological and computational sources for reliable protein-protein interactions

**DOI:** 10.1186/1471-2105-11-S7-S8

**Published:** 2010-10-15

**Authors:** Min Wu, Xiaoli Li, Hon Nian Chua, Chee-Keong Kwoh, See-Kiong Ng

**Affiliations:** 1 School of Computer Engineering, Nanyang Technological University, Singapore; 2 Institute for Infocomm Research, 1 Fusionopolis Way, Singapore; 3 Harvard University, 250 Longwood Avenue, SGMB-322 Boston, USA

## Abstract

**Background:**

Protein-protein interactions (PPIs) play important roles in various cellular processes. However, the low quality of current PPI data detected from high-throughput screening techniques has diminished the potential usefulness of the data. We need to develop a method to address the high data noise and incompleteness of PPI data, namely, to filter out inaccurate protein interactions (*false positives*) and predict putative protein interactions (*false negatives*).

**Results:**

In this paper, we proposed a novel two-step method to integrate diverse biological and computational sources of supporting evidence for reliable PPIs. The first step, interaction binning or InterBIN, groups PPIs together to more accurately estimate the likelihood (Bin-Confidence score) that the protein pairs interact for each biological or computational evidence source. The second step, interaction classification or InterCLASS, integrates the collected Bin-Confidence scores to build classifiers and identify reliable interactions.

**Conclusions:**

We performed comprehensive experiments on two benchmark yeast PPI datasets. The experimental results showed that our proposed method can effectively eliminate false positives in detected PPIs and identify false negatives by predicting novel yet reliable PPIs. Our proposed method also performed significantly better than merely using each of individual evidence sources, illustrating the importance of integrating various biological and computational sources of data and evidence.

## Background

Protein-protein interactions (PPIs) are at the core of the biological machinery of any living cells. PPIs are involved in almost every level of cellular functions, playing key roles in the transport machinery across the various biological membranes, the packaging of chromatin, the network of sub-membrane filaments, the regulatory mechanism of gene expression, and so on [1], while abnormal PPIs are key to various diseases. Research in PPIs will therefore allow the biologists to understand biological functions, uncover the underlying disease pathways, and provide the basis for new therapeutic approaches to benefit human beings [2].

In recent years, many high-throughput experimental techniques, most notably yeast-two-hybrid and anity purification, have been developed to enable comprehensive detection of PPIs. Numerous publicly available databases, such as DIP [3], BIND [4] and BioGrid [5], have been set up for researchers to access PPI data for biological and computational analysis.

However, the quality of current detected PPI data is far from satisfactory. The experimental conditions in which the detection methods are carried out may cause a bias towards detecting interactions that do not occur under physiological conditions. In other words, the experimental data may contain *false positive *PPIs that do not occur in the cell. At the same time, the high-throughput methods can also be biased against soluble or membrane proteins and fail to detect certain types of interactions such as weak transient interactions and interactions that require post-translational modification. This results in *false negative *detection and low experimental coverage of the interactomes [6,7].

How serious is the PPI data quality situation - specifically, what are the false positive and false negative rates in current PPI data? Many researchers have attempted to answer this question [6,8-11]. By comparing the overlap between the PPIs collected from different large-scale biological experiments, or checking the consistency of the cellular function or location information between the interaction partners, it has been found that the quality of the current PPI data is alarmingly low, with accuracy ranging from only 10% to 50% [6,11], and coverage lower than 50%. This is the case even for the most studied and curated interactome of Saccharomyces cerevisiae (yeast) [10]. One can expect the accuracy and coverage of the PPIs to be even lower for many other species.

Given the experimental limitations in high throughput PPI screenings, researchers have recently begun to exploit the growing availability of various additional biological and computational resources to address the issues on *false positives *and *false negatives *computationally. The computational methods can be categorized into two classes. The first line of bioinformatics research focuses on exploiting the topological properties of the PPI networks to assess the reliability of PPI. For example, Interaction Generality (IG1) [12] was proposed to detect false positives created by "sticky" proteins. IG2 [13] was subsequently proposed to measure the reliability of PPI using five pre-defined network motifs to dissect the local network topology. IRAP, or Interaction Reliability by Alternative Path [14], made use of the assumption that an interaction associated with an alternative path of reliable interactions is likely to be reliable, while PathRatio [15] measured the reliability of an interaction by considering all the alternative paths connecting these two target proteins. Several methods [16-18] were also proposed to quantify the propensity of two given proteins to be interacting partners based on the number of their common neighbors.

Another line of the research exploits the increasingly enriched genomic features to identify true positives and predict false negatives. Deane *et al. *[19] selected proteins pairs with similar gene expression profiles as true positives. Jansen *et al. *[20] employed a Bayesian network approach to combine genomic features such as gene expression profiles, co-essentiality, and co-localization, to predict novel interactions that were false negatives in the PPI networks. Kernel methods have also been used to detect false negatives [21,22]. Patil *et al. *[23] also used multiple genomic features, including sequence similarity, functional annotations and 3 D structures, to filter out false positives.

In this paper, we aim to exploit the use of both topological properties of PPI networks and the genomic features of proteins to identify reliable detected PPIs and predict putative interactions. We propose a method to integrate the diverse biological and computational resources. Our method consists of two steps: interaction binning (InterBIN) and interaction classification (InterCLASS). Since the diverse biological and computational evidences were individually scored in a different nature, we first group the protein pairs for each class of evidence into bins so that we can more accurately estimate the likelihood (Bin-Confidence score) that the protein pairs interact. Then, we employ machine learning methods such as support vector machines and Bayesian classifier to classify whether a detected or putative PPI is reliable or not using the Bin-Confidence score re-weighted evidences as features. We conduct experiments to show that our proposed method is significantly better than the existing methods, and that integrating the various biological and computational sources of data and evidence can provide better results.

### Our proposed framework

In this section, we present our two-step method for integrating diverse sources of biological and computational evidences for reliable PPI detection. First, we introduce each of individual biological and computational evidences which are used to assign original raw scores for protein pairs to indicate their propensity to interact. Then, we describe our InterBIN method to group protein pairs based on their scores from each evidence, and then define the more accurate Bin-Confidence scores for each protein pair. Finally, we describe our InterCLASS method that exploits the integration of the evidences re-weighted using Bin-Confidence scores and infers reliable PPIs using machine learning methods such as Support Vector Machines (SVM) and Bayesian classifier (BC).

### Individual biological and computational evidences

In this study, given a protein pair (*x*, *y*), we use each of the following biological and computational evidences to evaluate their propensity to interact. Each evidence *f *assigns a score *S*_*f *_(*x*, *y*) to (*x*, *y*) independently based on the nature by which the evidence was derived.

1. *Topological properties*. As we have discussed in the previous section, there are a number of topology-based methods to assess the reliability of protein interactions. PathRatio [15] and IRAP [14] assume that the protein interactions with more alternative paths are more reliable. The main difference between them is that PathRatio considers all the alternative paths while IRAP [14] only considers the strongest non-reducible alternative paths. The FS-weight [17], on the other hand, considers the number of common neighbors of the protein pair (*x*, *y*) -- if *x *and *y *interact with some common proteins (e.g, *z*), then (*x*, *y*) is assumed to share some physical or biochemistry characteristics that allow them to bind to these common proteins. The more common proteins they interact with, the higher chance they interact.

In this paper, we utilized all above three topological measures and compared their effectiveness to infer reliable protein interactions. In particular, for FS-weight, we used the following simplified variant as follows [17]:

(1) $$S_{FS}(x,\;y)=\frac{2\;\;\times \;\;|N_{x}\cap \;\;N_{y}|}{|N_{x}-N_{y}|+2\;\;\times \;\;|N_{x}\cap \;\;N_{y}|\;\;+\;\;1}$$

(2) $$\times \frac{2\;\;\times \;\;|N_{x}\cap \;\;N_{y}|}{|N_{y}\;\;-\;\;N_{x}|+2\;\;\times \;\;|\;\;N_{y}\cap \;\;N_{x}|+\;\;1},$$

where *N*_*x *_and *N*_*y *_are the set of direct neighbors of the protein *x *and *y *respectively.

2*. Gene expression profiles*. The correlation between the similarity of expression patterns for a pair of genes and interaction of the proteins they encode has been demonstrated for various species [24]. Statistical results have shown that protein pairs encoded by co-expressed genes interact with each other more frequently than with random proteins. This has allowed biologists to exploit large-scale gene expression to assess the reliability of protein interaction screens.

Given a protein pair (*x*, *y*), the proteins' propensity to interact can be measured by using the *Pearson Correlation Coefficient *between their encoded genes' expression profiles *G*_*x *_and *G*_*y *_as follows,

(3) $$S_{GE}(G_{x},\;G_{y})=\frac{\left|\sum_{i=1}^{n}(x_{i}-\bar{x})\;\;(y_{i}-\bar{y})\right|}{\sqrt{\sum_{i=1}^{n}{\left(x_{i}-\bar{x}\right)}^{2}}\sqrt{\sum_{i=1}^{n}{\left(y_{i}-\bar{y}\right)}^{2}}},$$

where *n *is the number of time points for the expression profiles and *x*_*i *_is the *i*^*th *^expression value of protein *x*'s expression profiles *G*_*x *_, whose average expression value is $\bar{x}$.

3. *Protein domains and their interactions*. Protein domains are evolutionarily conserved modules of amino acid sub-sequence that are postulated that as nature's functional "building blocks" for constructing the vast array of different proteins. Protein functional domains are thus regarded as essential units for such biological functions as the participation in transcriptional activities and other intermolecular interactions. The existence of certain domains in the proteins orchestrates the propensity for the proteins to interact due to the underlying domain-domain interactions. Databases, such as the Protein families (Pfam) database and others, have compiled comprehensive information about domains [25].

In this study, we use domain-domain interactions to evaluate the propensity of a protein pair to interact [26]. The propensity score between a protein pair (*x*, *y*) can be computed by the number of interacting domains that they contain:

*S*_*PF *_(*x*, *y*) = |{*d*_*i*_, *d*_*j*_)| *d*_*i *_∈*D*_*x*_, *d*_*j *_∈*D*_*y*_, *d*_*i *_interacts with *d*_*j*_}|, *D*_*x *_and *D*_*y *_are the set of Pfam domains of protein *x *and *y *respectively.

4. *Protein sequences and their similarity*. One of the most fundamental and successful tools in the field of bioinformatics is sequence alignment. By aligning protein sequences with each another, we can evaluate how similar the protein sequences are. In most cases, close homologs (> 30% sequence identity) physically interact with each other [27,28]. In particular, many of paralogs (close homologs within the same species) are known to interact with each other in heterodimer complexes. There many well-known examples such as the spliceosomes and many transcription factors [29]. Given two proteins *x *and *y*, the bit-scores from the BLAST results are used to show the sequence similarity between them, denoted as *S*_*SS*_(*x*, *y*).

In summary, the various biological and computational evidences mentioned above are widely-used for assessing the reliability of protein interactions. Another commonly-used biological evidence is functional similarity -- proteins that have the same molecular functions are more likely to interact since they are involved in the same biological processes. In this study, we intentionally exclude the use of functional information to predict reliable protein interactions -- we keep it to evaluate our experimental results eventually.

### InterBIN: grouping interactions by their original raw scores

The original raw scores obtained from each evidence, (e.g., FS-weight scores) have been directly exploited to identify reliable interactions previously [14,15]. However, since each of the evidences does not necessarily cover all interacting protein pairs as well as useful biological knowledge, it is necessary to integrate all these information to make our PPI prediction more robust.

Integrating original raw scores from various evidences is not as straightforward as one may think. Each piece of individual evidence actually provides information of a rather different nature; the original raw scores assigned by each method can differ significantly in scale, range, and distribution. For example, out of the 17262 interactions in DIP data, 9265 interactions (i.e., 53.7%) are with FS-weight scores less than 0.1 while 14099 interactions (i.e., 81.7%) are with IRAP scores at least 0.9. It is clearly not a good idea to integrate various evidences by directly using their original raw scores.

To address this issue, we re-weigh the evidence more robustly by estimating the likelihood that a protein pair (*x*, *y*) interacts given the observation that a evidence *f *assigns a score *S*_*f *_(*x*, *y*) to (*x*, *y*). To do this, we can examine all protein pairs that are assigned with the same score *S*_*f *_(*x*, *y*) in the training data and compute the fraction of those protein pairs that interact or occur in positive training set *P *(how to construct positive and negative training data *P *and *N *will be described in next subsection). However, the scores assigned by each evidence *f *may not be discrete, and there may not be enough protein pairs that are assigned the same score. Thus, it is necessary that we first group the protein pairs with similar scores together.

Let us now introduce how to group interactions and calculate the Bin-Confidences for them using our method InterBIN. For a evidence *f*, let *S*_*f *_(*x*, *y*) be the original raw score of the protein pair (*x*, *y*).

Assuming that there are *n *protein pairs, they can be divided into different Bins based on their original raw scores in following simple steps.

1. Sort all protein pairs based on their original raw scores.

2. For the sorted protein pairs, (*x*_1_, *y*_1_), ..., (*x*_*n*_, *y*_*n*_), first *μ *protein pairs (i.e., (*x*_1_, *y*_1_), ..., (*x*_*μ*_, *y*_*μ*_)) are inserted into the first group $G_{1}^{f}$. All the protein pairs that have the same score as the protein pair (*x*_*μ*_, *y*_*μ*_) will be also inserted into $G_{1}^{f}$ Let the size of $G_{1}^{f}$ be (*μ *+ *k*).

3. Start from the protein pair (*x*_μ + k + 1_; y_μ + k + 1_) and repeat Step 2 until *m *groups are obtained finally. If the last group $G_{m}^{f}$ has the size smaller than *μ *(Here, *μ *is an integer to represent the group size. To better show our experimental results, we have used the relative group size, *μ*/*n*, to replace in our experiment section, where *n *is the total number of protein pairs in the training set), it is merged into the group $G_{m-1}^{f}$.

Using the above method, we divide all the protein interactions into different groups such that the interactions within the same group have similar original raw scores and each group also has similar size. We can then estimate the confidence score for each group by the proportion of positive examples within it. Basically, the more positive protein interactions a group $G_{k}^{f}$ has, the more reliable a protein interaction within the group $G_{k}^{f}$ is. For a protein pair (*x*, *y*) in $G_{k}^{f}$, we re-weigh it with a score, denoted as *Bin-Confidence*_*f *_(*x*, *y*) in equation 4, which is the confidence score of $G_{k}^{f}$.

(4) $$Bin-Confidence_{f}(x,\;y)=\frac{\left|\{(x_{i},y_{i})|(x_{i},y_{i})\in P,(x_{i},y_{i})\in G_{k}^{f}\}\right|}{\left|G_{k}^{f}\right|},$$

where *P *is the positive training set.

### InterCLASS: integrating evidences by machine learning techniques

For all the protein pairs in a detected PPI network, InterBIN basically transforms their original scores from various evidences into standard Bin-Confidence scores. Next, we show how we incorporate the Bin-Confidence scores to build classifiers to identify reliable interactions.

In order to use machine learning to build an accurate PPI classifier, first, we need to compile a positive and a negative training set *P *and *N*. While experimentally derived protein interaction data are publicly available in a number of databases such as DIP [3] and BioGrid [5] etc, we need to be careful in the selection of positive and negative examples due to their noisy nature (as discussed in Introduction previously). In this study, we select a protein pair as a positive example only if it was detected by small-scale experiments (not high throughput screenings) or by multiple (at least 3) wet-lab techniques. As for constructing a negative training set, we adopt the method used in [23] by considering those (potentially wrongly detected) protein pairs in the PPI databases whose two interacting partners are from different cellular locations. This is because proteins located in the different cellular components are unlikely to interact physically. We then consider all the other protein pairs in the PPI dataset (that do not belong to *P *and *N *) to be with an unknown status -- we will decide if they interact using our machine learning-based classifiers discussed below.

Given the positive and negative training sets *P *and *N*, for each protein pair (*x*, *y*) in *P *and *N*, we gather the original score *S*_*f *_(*x*, *y*) for each evidence *f*. Next, we compute the *Bin-Confidence*_*f *_(*x*, *y*) for all the protein pairs (*x*, *y*) with respect to *f*. With them, we are now ready to build the final classifiers that integrate the diverse biological and computational evidence sources. In this study, we apply two different types of classifiers, namely, Support Vector Machines and Bayesian classifier, that are popular classifiers in many bioinformatics applications.

#### Support vector machines

The SVM [30] is a state-of-the-art classification technique in machine learning and it has been proven to be one of the best classifiers in many application domains such as text categorization, image recognition, protein remote homology detection, and so on. The basic principle of SVM is to solve the convex optimization problem that finds a maximum-margin hyperplane. The hyperplane can then be used to classify new unseen samples as positive or negative class.

For each training example in *P *and *N*, we can build a feature vector (*f*_1_, *f*_2_, ..., *f*_*λ*_) where *f*_*i*_(*i *= 1, 2, ..., *λ*) is a score (either the original raw score, or the re-weighted Bin-Confidence computed by InterBIN) from the individual evidences where is the total number of biological evidences. In this way, SVM is able to train a classifier [31] exploiting all the evidences together to classify those detected protein pairs with unknown status.

#### Bayesian classifier

Given a protein pair (*x*, *y*) and its *n *features *f*_1_, ..., *f*_*λ*_, the probability that it is a negative interaction is denoted as *P*{*false|f*_1_, ..., *f*_*λ*_}. We can assume that the *λ *features of a protein pair are conditionally independent and then apply Bayesian rule to calculate this probability. In this way, the probability of (*x*, *y*) to be positive, *P*{*true|f*_1_, ..., *f*_*λ*_}, can be calculated as follows:

(5) $$\begin{array}{cc} P\{true|f_{1},\;\ldots ,\;f_{\lambda }\} & =1-P\{false|f_{1},\;\ldots ,\;f_{\lambda }\}\\ & =1-\prod_{i=1}^{\lambda }(1-P\{true|f_{i}\}) \end{array},$$

where the probability of (*x*, *y*) to be positive with respect to the feature *f*_*i*_, *P*{*true|f*_*i*_}, can be estimated by using either its original raw score or its Bin-Confidence score for *f*_*i*_.

Basically, for a given protein pair, the Bayesian method will give it a high reliability score if there are certain evidences that show that it is a positive interaction.

## Results and discussions

We have performed comprehensive experiments to evaluate the proposed techniques. Since our method used Bin-Confidence scores instead of the original raw scores, in this section, we first show the benefits of using Bin-Confidence scores. Then, we show that our integrating method is very effective in identifying false negatives and false positives than existing methods.

### Comparison between Bin-Confidence scores and original raw scores

Linear SVM as well as SVM with polynomial kernels and Gaussian kernels (using SVM^*light *^software [31]) have been used in our experiments. Since they have comparable performance, we only focused on presenting the results from the linear SVM in this paper. For easy reference, let us call our integrative method using SVM and Bayesian Classifiers InterSVM and InterBC respectively.

After collecting the positive and negative training examples, we calculated the Area Under ROC Curve (AUC) for each method using both the original raw scores and Bin-Confidence score. The AUC for each method was obtained by performing a 5-fold cross validation. To group protein pairs and calculate their Bin-Confidence score, *μ *was set as 1% on DIP data and 0.5% on BioGrid data. For example, *μ *= 1% means that each group has at least *n *× 1% protein pairs and *n *is the total number of protein pairs in the training set. We will explain how such a parameter setting came about in the coming subsection.

Table 1 shows the AUC of each method using original raw scores and Bin-Confidences, respectively (the running time for PathRatio to perform on comprehensive BioGrid data was prohibitively high. Thus, we were unable to obtain the results of PathRatio on BioGrid data). From the results on the two different PPI datasets, we can draw following two conclusions. First, integrating diverse individual evidences can improve the prediction accuracy. For example, FS-weight is the best-performed individual feature on DIP data, whose AUC is 0.768, while InterSVM and InterBC with Bin-Confidences have an AUC of 0.804 and 0.787, which are 3.6% and 1.9% higher than that of FS-weight respectively. Second, while grouping protein interactions and calculating Bin-Confidence scores may not improve each original individual method very much, it improves the accuracy of InterSVM and InterBC. As shown in Table 1, the AUC of InterBC with Bin-Confidence scores is 0.782 on DIP data and 0.750 on BioGrid data, which is 7.7% and 15.0% higher than that of InterBC using the original raw scores respectively, indicating that our InterBIN grouping technique makes our integrative approach more robust.

**Table 1:** AUC of each method using original raw scores and Bin-Confidences, respectively.

Methods/Data	DIP data		BioGrid data	
	
	Original Raw Scores	Bin-Confidence	Original Raw Scores	Bin-Confidence
FS-weight	0.741	0.768	0.741	0.745
PathRatio	0.702	0.710	-	-
IRAP	0.686	0.723	0.580	0.594
Gene-expression Correlation	0.549	0.560	0.580	0.566
Interacting Domains	0.547	0.544	0.561	0.562
Sequence Similarity	0.576	0.569	0.523	0.529
InterSVM	0.776	0.804	0.749	0.768
InterBC	0.710	0.787	0.600	0.750

It is interesting to note that the genomic evidences appeared to have much worse performance than the topological evidences in Table 1. One reason is that the original raw scores from the genomic evidences are too limited in coverage. For example, in DIP data, only 5663 out of 17262 interactions, i.e., 32.8%, have non-zero original scores from the protein domain information, while majority of the interactions (16036 or 92.9%) have non-zero IRAP scores.

### Effect of the parameter μ

**Figure 1:**
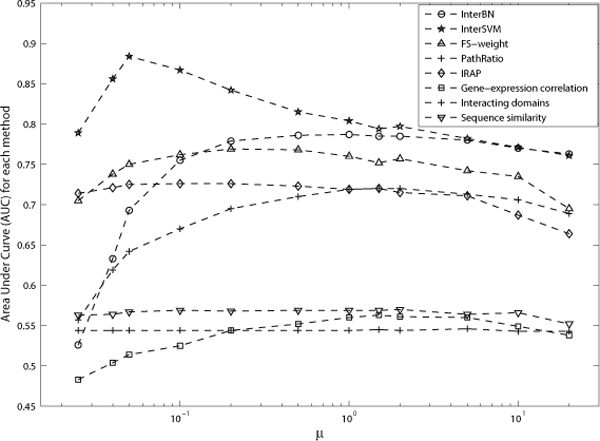
**AUC for each method as the parameter *μ *varies on DIP data**. Figure 1 shows the AUC for each method as the parameter *μ *varies on DIP data.

**Figure 2:**
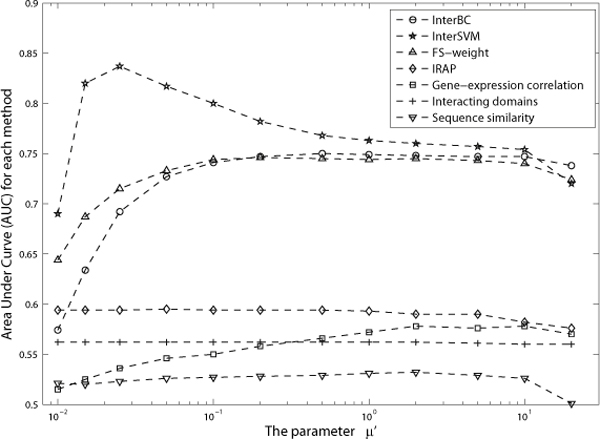
**AUC for each method as the parameter *μ *varies on BioGrid data**. Figure 2 shows the AUC for each method as the parameter *μ *varies on BioGrid data.

The parameter *μ*, which was used to determine the group size, has a direct effect on the Bin-Confidences and on the accuracy for methods using Bin-Confidences. Figure 1 and 2 show the AUC for each method on DIP data and BioGrid data respectively as *μ *varies.

A larger *μ *generally means a smaller number of groups. Therefore, there will be more interactions in each group having the same Bin-Confidence. As such, the resulting Bin-Confidence assignments can become too general as the large number of interactions within the same group cannot be distinguished. On the other hand, using a smaller *μ *risks getting less accurate Bin-Confidences due to the small number of samples in each group. Figure 1 and 2 confirms that group sizes that are either too large or too small will result in poor accuracy for each method.

With the DIP dataset, the individual evidences as well as InterBC achieved their decent AUC when *μ *was around 1%, while InterSVM achieved its highest AUC when *μ *was set as 0.05%. However, since there are only 8206 positive and negative examples in the DIP dataset, each group would contain only around 4 interactions if *μ *were to be set as 0.05%, resulting in inaccurate Bin-Confidences for interactions and the over-fitting of the SVM. As such, we recommend that *μ *is set as 1% for DIP data, and *μ *is set as 0.5% for BioGrid data.

### False positive/negative detection

Note that while protein-protein interactions have been detected and stored in the protein interaction databases such as DIP [3] and BioGrid [5] etc, many of them may not be reliable (i.e., with an unknown status that is neither in *P *nor in *N *). We can apply the classifiers learnt from the training data to classify those with unknown status to address the false positive detection issue.

Meanwhile, although current PPI data are becoming more and more comprehensive, it is still far from complete due to inherent experimental limitations and biases. Recent paper [32] concluded that false negatives is a serious issue in experimental detection of protein-protein interactions -- the false negative rate of yeast two-hybrid experiments (which is currently the most widely used technique that detects in vivo pairwise interactions) to be around 50%. Given the generalization capability of the classifiers learnt from the training sets, they can also be used to detect false negative interactions by predicting putative interaction between undetected pairs of proteins. In this paper, to avoid classifying all the possible undetected protein pairs for false negative elimination, we generate potential false negative candidates using those protein pairs with at least 2 common neighbors in the DIP database [33].

**Figure 3:**
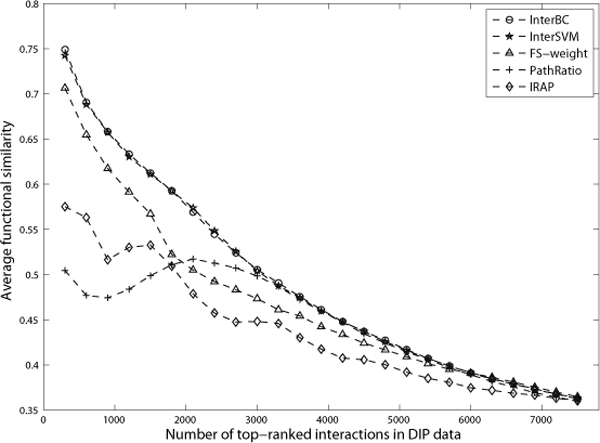
**The average functional similarity of top-ranked interactions in DIP data**. Each interaction may have functional similarity score which is the semantic similarity of GO terms annotating these two interacting proteins. Figure 3 shows the average functional similarity of top-ranked interactions in DIP data.

**Figure 4:**
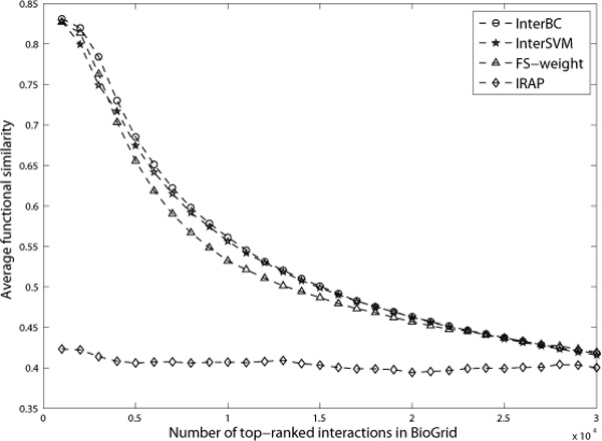
**The average functional similarity of top-ranked interactions in BioGrid data**. Figure 4 shows the average functional similarity of top-ranked interactions in BioGrid data.

**Figure 5:**
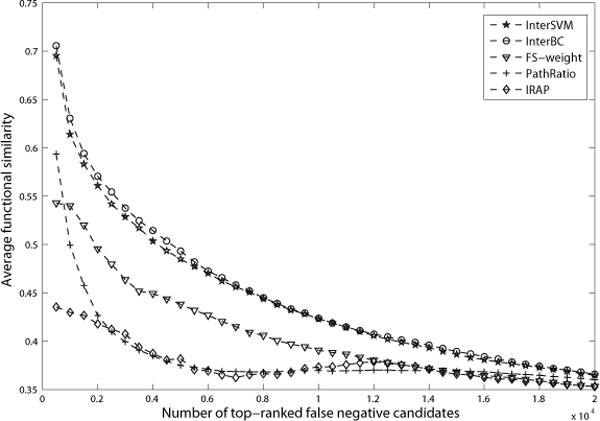
**The average functional similarity of top-ranked false negative candidates generated from DIP data**. In DIP data, protein pairs with at least 2 common neighbors were selected as false negative candidates, resulting in 33482 such candidates. Figure 5 shows the average functional similarity of the top-ranked false negative candidates.

We have applied our integrative methods which is learned from existing reliable interactions and non-interactions, to predict false negatives and false positives. The quality of the predicted false negatives and false positives is evaluated and validated by the corresponding functional similarity scores between the protein partners. Figure 3, 4 and 5 show the average similarity scores for top-ranked interactions (or predicted interactions). A point (*x*, *y*) in these figures means that top-*x *interactions (or predicted interactions) have an average similarity *y*.

#### False positive detection

For those interactions in the PPI datasets that we have labeled with an unknown status, we rank them by their decision values from each classifier. For DIP data, the top-ranked interactions by InterBC and InterSVM were shown to have much higher functional similarity than those ranked by individual evidences, including FS-weight, PathRatio and IRAP. (Genomic features here, such as sequence similarity, gene expression correlation and interacting domain information, have poor performance as shown in Table 1.

Therefore, we did not present the functional similarity for interactions ranked by these evidences in figures 3, 4 and 5.) Therefore, those detected interactions that have higher ranks by InterSVM or InterBC are more likely to be true positives. Conversely, the detected interactions with lower ranks by InterSVM or InterBC tend to have lower similarity and are thus likely to be false positives.

In the BioGrid dataset, the overall performance of InterSVM and InterBC is still slightly better than that of FS-weight, as shown in figure 4. However, the top 3000 interactions ranked by FS-weight have even higher functional similarity than those by InterSVM. This is due to the fact that IRAP suffers from a poor performance in BioGrid data as shown in table 1 and figure 4. As such, it is understandable that our integrative method cannot improve the performance much as it is also penalized by including those evidences with very poor performance.

#### False negative detection

It is very popular to take those protein pairs with at least a common neighbor as false negative candidates [34]. To avoid generating too many false negative candidates in those large PPI networks, we selected those protein pairs with at least 2 common neighbors as candidates in this work. In DIP data, we finally collected 33,482 such candidates.

Figure 5 shows the average functional similarity of the top-ranked false negative candidates. The top-ranked candidates by InterSVM and InterBC are shown to have much higher functional similarity than those by FS-weight, PathRatio and IRAP. Thus, they are more likely to be novel interactions that are false negatives in the original datasets. In Figure 5, the average functional similarity of the top 2000 candidates by InterSVM and InterBC are 0.561 and 0.571 respectively -- both are even higher than that of all interactions in our gold standard benchmark DIP core data (detected by small-scale experiments; all the protein interactions in the core have an average functional similarity 0.545), illustrating that our integrative approach has truly good results in predicting novel yet reliable protein interactions for eliminating false negatives in current PPI datasets.

We further analyzed the two sets of top 2000 ranked candidate interactions (from DIP) by InterSVM and InterBC respectively and found that they actually have a big overlap--there are 1574 interactions in common. The top 2000 candidates ranked by InterSVM also have 476 interactions that are confirmed to be reliable interactions in BioGrid data, while the top 2000 ranked by InterBC have 462 interactions that are similarly reliable. For example, the interaction between proteins YCR081W and YDR443C in both above two sets is believed to be reliable as it has been detected multiple times by TAP-MS [35-37]. The high overlap between the predicted sets, as well as their large and almost equal numbers of confirmed interactions further arm the usefulness of our integrative method for false negative elimination.

### Comparison with Patil's integration method

Given an interaction, Patil *et al. *[23] proposed a naive bayesian method to check whether it is reliable by integrating following three features: (1) whether this interaction has homologous interactions, *i.e*., these two interacting proteins have homologs that interact in other species; (2) whether interacting proteins have at least one GO annotation in common and (3) whether interacting proteins have interacting Pfam domains. In our proposed method, we have utilized six commonly-used features to identify reliable interactions. Please note that GO annotations (the second feature) typically are not used for predicting protein interactions since they are reserved to evaluate the performance of computational/experimental methods. In order to compare with Patil's method, we also included two new features in our method, namely, the number of homologous interactions and the semantic similarity of two interacting proteins (measured by the semantic similarity of their GO annotations as show in equation 6).

Table 2 shows the AUC of each method (using 5-fold cross validation) on DIP data and BioGrid data, respectively. The performance for InterBC using original scores on two datasets is worse than that of Patil's method. However, it outperforms Patil's method when it exploits Bin-Confidence scores.

**Table 2:** The comparison between Patil's method and ours.

Methods/Data	DIP data		BioGrid data	
**Patil's method**	**0.810**		**0.784**	
	
	**Original Raw Scores**	**Bin-Confidence**	**Original Raw Scores**	**Bin-Confidence**
	
InterSVM	0.852	0.865	0.797	0.818
InterBC	0.743	0.841	0.776	0.798

Meanwhile, InterSVM with either original scores or Bin-Confidence outperforms Patil's method on both datasets. We believe that the reasons why our proposed methods perform better than Patil's method are as follows. First, we integrated more biological and computational features that are indeed beneficial, e.g., FS-weight [14]. Second, Bin-Confidences as shown in both table 1 and 2 can effectively enhance the performance of the classifiers. Third, as each feature value in Patil's method is binary (either 0 or 1), our probabilistic-based methods with fine granularity can better exploit each individual feature for identifying reliable interactions. Take homologous interactions as an example, Patil's method only cares whether an interaction has homologous interactions or not. However, our methods care how many homologous interactions a given interaction has in other species, because an interaction with more homologous interactions would be considered to be more reliable in our methods.

## Conclusions

Protein-Protein interactions play important roles in cellular processes and provide an invaluable resource for network-based biological knowledge discovery such as protein complex detection [38], protein function prediction [28], and disease pathway discovery [2]. While high-throughput experimental techniques seemed to provide us with many large-scale PPI datasets, their usefulness is diminished by high level of noise and incompleteness. To eliminate the abundant false positives and false negatives in the current PPI datasets, it is important to develop robust methods for identifying and predicting reliable interactions.

In this paper, we integrate diverse sources to detect PPI for eliminating the false positives and negatives. We first estimate the Bin-Confidences for interactions by grouping them based on their original raw scores from each source. Using the robust Bin-Confidence scores of protein pairs from the various sources as features, we train machine learning models using SVM and BC to integrate the sources and construct classifiers to detect reliable interactions. Experimental results show that our integrative method outperforms the approaches merely using each of individual topological or genomic evidences. The accuracy of SVM and BC is also significantly improved by using the Bin-Confidences instead of the original raw scores.

Since our Bin-based machine learning approach is designed to integrate diverse biological and computational evidences, as future work, we will collect more relevant biological data (such as their purification records, the mass spectrometry scores, and so on), as well as computational evidences (such as phylogenetic similarity) to be included in our method. Based on the current results, we are confident that the inclusion of these additional evidences, while diverse in nature, will allow us to effectively filter false positives and predict novel false negatives from the current PPI datasets.

## Methods

We will first introduce the experimental data used in our work. We will also introduce evaluation measures for the accuracy of various prediction methods. The validation for the identified false negatives and false positives using Gene Ontology terms will also be presented.

### Experimental Data

Two publicly available yeast PPI data, DIP [3] and BioGrid [5], were downloaded and used in our experiments. After removing the self-interactions, DIP consists of 17262 interactions and BioGrid consists of 71020 interactions. To train classifiers, we selected the positive training examples as follows. First, since all the 4314 interactions in DIP-core [3] are detected by small-scale experiments or by multiple wet-lab techniques, they are regarded as positive examples for the DIP data. Second, 13424 interactions in BioGrid which (1) are detected by more than 3 experiments, or (2) are in DIP-core, or (3) are high-quality interactions in Krogan et al.'s purification data [36] or Gavin et al.'s data [35], are selected as positives. We selected the negative training examples as described previously -- known interactions whose two interacting partners are observed with different cellular locations are considered as negatives [23]. Specifically, given two interacting proteins, the interaction between them is regarded to be negative if the semantic similarity of their GO terms (Cellular Component) is below a certain threshold. In our experiments, semantic similarity between GO terms are calculated by the method in [39] and the threshold is set as 0.4 [22]. We have selected 3892 negative examples for DIP data and 11557 negatives for BioGrid data respectively.

As to the genomic sources, interacting domains were downloaded from following 3 databases, 3DID [40], iPfam [41] and DOMINE [42]. Gene-expression data were downloaded from [43], while Gene-Ontology data were downloaded from [44], and sequence data were downloaded from UniProt (the Universal Protein Resource, http://www.uniprot.org/).

### Receiver Operating Characteristics

The Receiver Operating Characteristics (ROC) curve is a graphical plot of the sensitivity vs. 1-specificity for a classifier as the decision threshold varies. The Area Under the ROC Curve (AUC) is a widely-used measure of the accuracy of a specific classifier.

In this paper, in addition to our integrative approach (using SVM and BC), each original method for identifying reliable interactions can also be regarded as a classifier. For example, IRAP [14] can be used a classifier -- both the original IRAP score and the Bin-Confidence score for each protein pair can be considered as a decision value by the IRAP classifier. In other words, each original method for the various evidences can also have an AUC to show its accuracy.

### Validation of False Positives/Negatives

Since a protein performs a certain cellular function by interacting with other proteins, the cellular functions of interacting proteins are likely to be similar. Functional similarity between proteins based on the GO terms are often exploited as computational validation of protein-protein interactions [14,15]. Assuming that each protein pair can be computed with a score which is the functional similarity between its two interacting partners, protein pairs with top decision values under a better classifier are expected to have higher average similarity scores.

Given two proteins *x *and *y *annotated by {*g*_11_, ..., *g*_1*m*_} and {*g*_21_, ..., *g*_2*n*_} respectively, their functional similarity defined in [39], *S*_*GO*_(*x*, *y*), is calculated as follows.

(6) $$S_{GO}(x,\;y)=\frac{\sum_{1\leq i\leq m}\underset{1\leq j\leq n}{\max}\;sim(g_{1i},g_{2j})+\sum_{1\leq j\leq n}\underset{1\leq i\leq m}{\max}\;sim(g_{1i},g_{2j})}{m+n}$$

where *sim*(*g*_1*i*_, *g*_2*j*_) is the semantic similarity between GO terms *g*_1*i*_and *g*_2*j*_defined in [39].

## Competing interests

The authors declare that they have no competing interests.

## Authors' contributions

MW, XL and HNC conceptualized and designed the method and drafted the manuscript together. MW was responsible for the implementation. CKK and SKN participated in discussion and conceptualization as well as revising the draft. All authors read and approved the manuscript.
